# Glow flow ionization mass spectrometry of small molecules. A comparison of a glow flow ionization source (‘GlowFlow’) with electrospray ionization and atmospheric pressure chemical ionization

**DOI:** 10.1002/rcm.9327

**Published:** 2022-06-15

**Authors:** Rhodri N. Owen, Stevan Bajic, Steven L. Kelly, Michael R. Morris, A. Gareth Brenton

**Affiliations:** ^1^ Institute of Life Science, Faculty of Medicine, Health and Life Science Swansea University Swansea UK; ^2^ Waters Corp Wilmslow UK

## Abstract

**Rationale:**

Ionization by atmospheric pressure gas discharge has been employed for a long time in mass spectrometry. Inductively coupled plasma mass spectrometry is an exemplar, and widely used for elemental analysis. The technique has less uptake in organic mass spectrometry. We describe a simple source design that can be readily implemented in most atmospheric pressure ionization (API) systems and compare its performance with that of electrospray ionization (ESI) and atmospheric pressure chemical ionization (APCI).

**Methods:**

An in‐house designed helium gas discharge source (referred to as ‘GlowFlow’) was used on a Xevo G2‐S time‐of‐flight mass spectrometer. The GlowFlow source was transferred to a compatible Xevo TQ‐S triple‐quadrupole mass spectrometer using an ultrahigh‐performance liquid chromatograph inlet. Its performance was compared to that of Waters ESI and APCI sources.

**Results:**

Preliminary results of GlowFlow on the Swansea instrument are presented to establish context and include analysis of low‐molecular‐mass polymers, benzoic acid and cinnamic acid. Comparison of performance on the Xevo TQ‐S triple‐quadrupole mass spectrometer involved three test mixtures. The method limits of detection (six‐mix) for positive‐ion GlowFlow source were between 0.03 and 10.00 pg with good linear response over two to four orders of magnitude and values of *R* ^2^ > 0.98. The GlowFlow ionization source provided a signal intensity that was an order of magnitude greater than that of ESI for an atmospheric pressure gas chromatography standard mix and ionized several compounds that ESI could not.

**Conclusions:**

The current GlowFlow design is relatively simple to retrofit to most API systems due to its small size. The sensitivity of the GlowFlow design is typically an order of magnitude less than that of ESI in positive‐ion mode, but similar in sensitivity in negative‐ion mode and comparable to that of APCI.

## 1 INTRODUCTION

Glow flow ionization (GFI) is a term describing atmospheric pressure ionization (API) based on a gas discharge where helium is typically used. We propose the acronym ‘GFI’ can be used to define an array of similar techniques (some discussed below) used in analytical mass spectrometry. A recent GFI design from our group,^1^, ^2^ which we refer to as ‘GlowFlow’, incorporates a compact and simple construction that is suitable for analytical mass spectrometry. This GFI source can be readily retrofitted to most API systems with minimal modifications. A comparison study was undertaken, using a Xevo TQ‐S triple‐quadrupole mass spectrometer based at the Waters' research group (Waters Corp., Wilmslow, UK), between our GlowFlow source, an electrospray ionization (ESI) source and an atmospheric pressure chemical ionization (APCI) source.

Our earliest GFI designs followed the work of Hieftje and co‐workers^3^ (flowing atmospheric pressure afterglow, FAPA), where a remote discharge cell feeds a helium afterglow onto the sample, in a gaseous, liquid or solid form.^4^ At that time our laboratory ran thousands of non‐polar samples every year by electron ionization and atmospheric solids analysis probe.^5^ Thus, we were motivated to search for techniques that could handle compounds, from non‐polar to polar chemistries, using a single method. Atmospheric pressure glow discharge (APGD) ionization seemed a prospect to study for that venture. The GlowFlow design is simple and can analyse wide ranging chemistries whilst displaying good analytical figures‐of‐merit, particularly in negative‐ion mode.

Ionization mechanisms occurring in APGD have been described previously^6^ and involve excited metastable helium (He*, 19.8 eV) and helium ions (He^+^, He_2_^+^) which subsequently react with background atmospheric gases such as nitrogen, oxygen and water vapour to create reagent species, e.g. H_3_O^+^ (positive mode) and OH^−^ (negative mode), leading to sample ions of the types [M + H]^+^, [M − H]^−^ and M^+•^. More complex molecule ion species can arise via substitution reactions such as oxidation leading to ions of the type [M − H + O]^+^ in the case of hydrocarbons.^1^, ^6^ In the literature, there have been several gas discharge techniques reported, often using argon gas and with some aimed at lightweight portable mass spectrometry applications. Of note in the literature are those for surface analysis,^7^ analytical mass spectrometry,^8^ microplasma‐based FAPA,^9^ a mini‐FAPA source coupled to capillary electrophoresis,^10^ differentiation of functional isomers^11^ and the helium plasma ionization designs of Pavlov et al.^12^, ^13^ The latter design seems very promising for GFI applications as it incorporates a dopant bleed into the analyte flow providing *inter alia* a source of protons and reagent species to enhance ionization. These sources are based on APGD where the analyte is directly exposed to the glow discharge region. In contrast, the direct analysis in real time source^14^ uses a helium, argon or nitrogen corona discharge in combination with an exit electrode that is biased to remove electrons and negative ions, or alternatively positive ions, from the discharge, whilst preserving the metastable neutral species. The GlowFlow source, operated in positive‐ion mode, has consistently given weaker signal than electrospray in earlier measurements at Swansea. This suggests a deficit of proton‐donating reagents (e.g. H_3_O^+^) which could arise as the nebulizer gas and GFI source, in that instrument, employ pure nitrogen and helium, and thus the source is operating in an environment deprived of protons. A similar dopant approach has been used successfully in atmospheric pressure photoionization sources.^15^, ^16^

**FIGURE 1:**
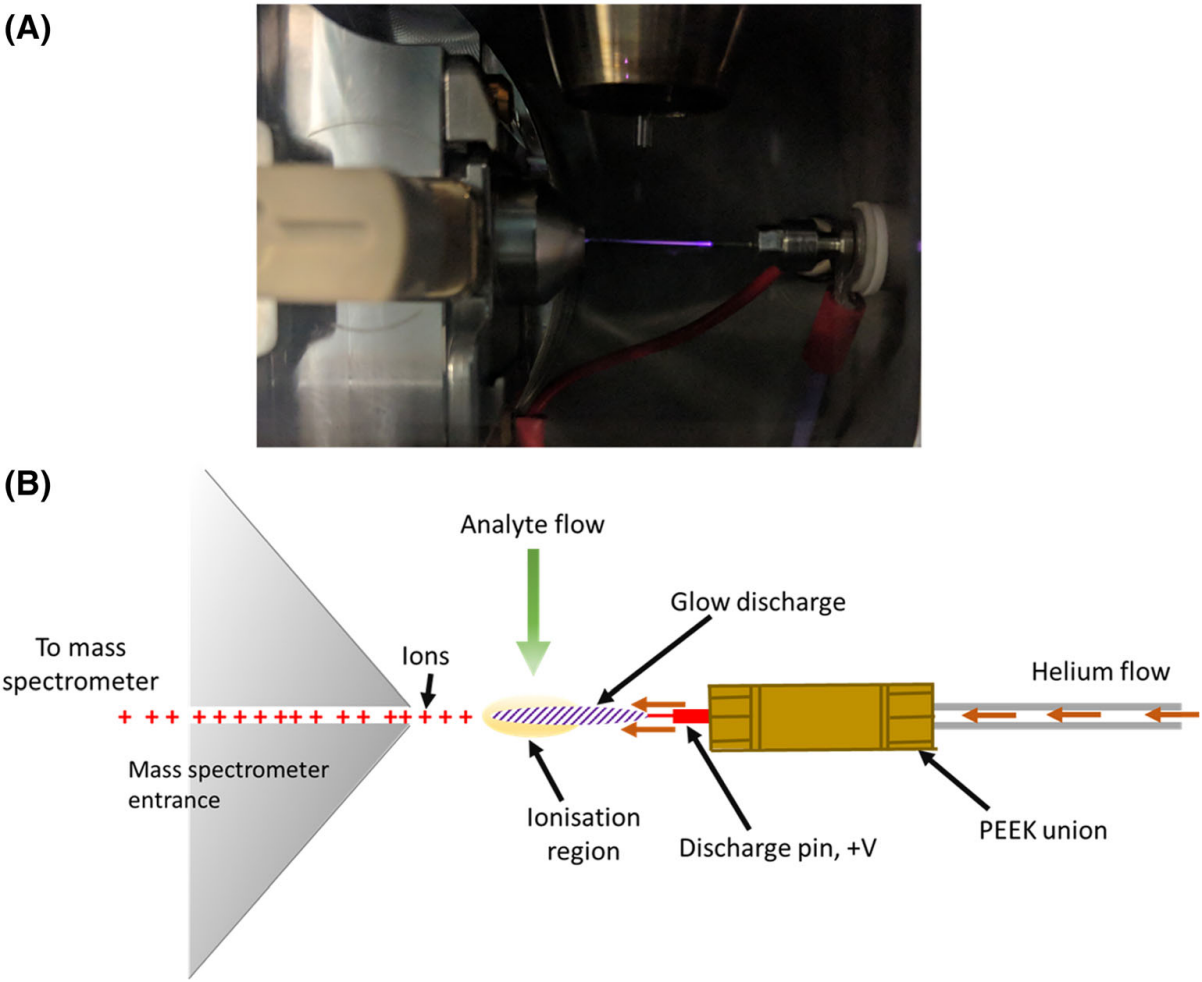
(A) Image of the GlowFlow system in a Waters Xevo G2‐S universal source housing. The ionization source is to the right, and axially opposite the mass spectrometer entrance (left). A heated nebulizer connected to an LC system is employed to desorb samples, as shown at the top centre. (B) Illustration of the GlowFlow design. Helium gas flows into a PEEK union where a gas discharge is formed downstream of a stainless steel electrode. The discharge region is arranged to cross the analyte flow [Color figure can be viewed at wileyonlinelibrary.com]

In recent GFI studies we examined a range of sample types that involved several ionization mechanisms.^1^, ^2^ Here we briefly introduce four further GFI examples for context: polymers, LC/MS studies, benzoic acids and atmospheric pressure gas chromatography (APGC) standards; however, the main thrust of the article is the comparison of GlowFlow with ESI and APCI on a triple‐quadrupole instrument. A Xevo TQ‐S mass spectrometer was chosen as our GlowFlow source could be directly fitted to the triple‐quadrupole system as the housing, mechanical, gas and electrical connections are all common.^17^ The study was carried out on well‐established test mixtures in positive‐ and negative‐ion modes. Ultrahigh‐pressure liquid chromatography data were also obtained to verify the chromatographic fidelity of the GlowFlow arrangement (but not included herein). The samples were delivered via an integrated APCI IonSABRE II (heated nebulizer) probe which was positioned perpendicular to the GFI source and mass spectrometer inlet, as shown in Figure 1.

The addition of ‘dopants’ to enhance ionization is well documented: from prior sample derivatization in electron ionization, to more recent examples where the dopant is added directly to the ionization region such as in atmospheric pressure photoionization^15^, ^16^ and the extensive work of Trimpin.^17^ The GlowFlow study using the triple quadrupole did not include dopant studies, due to limited time.

## 2 EXPERIMENTAL

### 2.1 Chemicals

Swansea University: High‐purity 3‐nitrobenzoic acid, 2,5‐dihydroxybenzoic acid, 4‐fluorobenzoic acid, pentafluorobenzoic acid, pentafluorocinnamic acid, polyethylene glycol (PEG 400), polypropylene glycol (PPG 725) and polyethylene imine (PEI) were purchased from Merck (Gillingham, UK).

Waters Research Group (Wilmslow, UK): Samples six‐mix (p/n: 186006861), APGC reference standard (p/n: 700005254‐6) and E&L screening standard (p/n: 186008063) were supplied by Waters (Wilmslow, UK); refer to the supporting information for further details on these three standard mixtures.

### 2.2 Mass spectrometer

Swansea instrument: data were acquired on a Xevo G2‐S time‐of‐flight mass spectrometer (Waters Corp., Wilmslow, UK) fitted with our prototype GlowFlow source. The GlowFlow system is described elsewhere^2^ and the source can be fitted with an ESI probe or a heated nebulizer APCI probe (<550°C).

Waters instrument: a Xevo TQ‐S triple‐quadrupole mass spectrometer located in Wilmslow (Waters, UK) and fitted with GlowFlow, ESI and APCI sources.

Data were obtained and processed using MassLynx 4.1 (Waters Corp., Wilmslow, UK) and the R statistical software package.^18^

### 2.3 High‐performance liquid chromatography (HPLC) equipment and conditions

Swansea instrument: a 1100 Series HPLC liquid chromatograph (Agilent Technologies, Stockport, UK). A C_18_ 3 μm, 2.1 × 150 mm column (Fortis Technologies Ltd, Cheshire, UK). Solvent A was water with 2% acetonitrile and 0.2% acetic acid, and solvent B was acetonitrile with 0.2% acetic acid. A linear solvent gradient was used with 0% solvent B at 0 min and increasing to 95% solvent B at 20 min at a flow rate of 0.2 mL min^−1^.

Waters instrument: a Waters Acquity UPLC I‐Class liquid chromatograph. An Acquity BEH C_18_ 1.7 μm, 2.1 × 50 mm column (Waters, Wilmslow, UK) was used. For the E&L screening standard,^19^ a CORTECS UPLC C_18_ 1.6 μm, 2.1 × 100 mm column (Waters, Wilmslow, UK) was used.

## 3 RESULTS AND DISCUSSION

The work reported in Sections 3.1 to 3.3 was conducted at Swansea University, Faculty of Medicine, whereas the work reported in Sections 3.4 and 3.5 was conducted at the Waters mass spectrometry research facilities (Wilmslow, UK).

### 3.1 Analysis of low‐molecular‐mass polymers by GlowFlow

**FIGURE 2:**
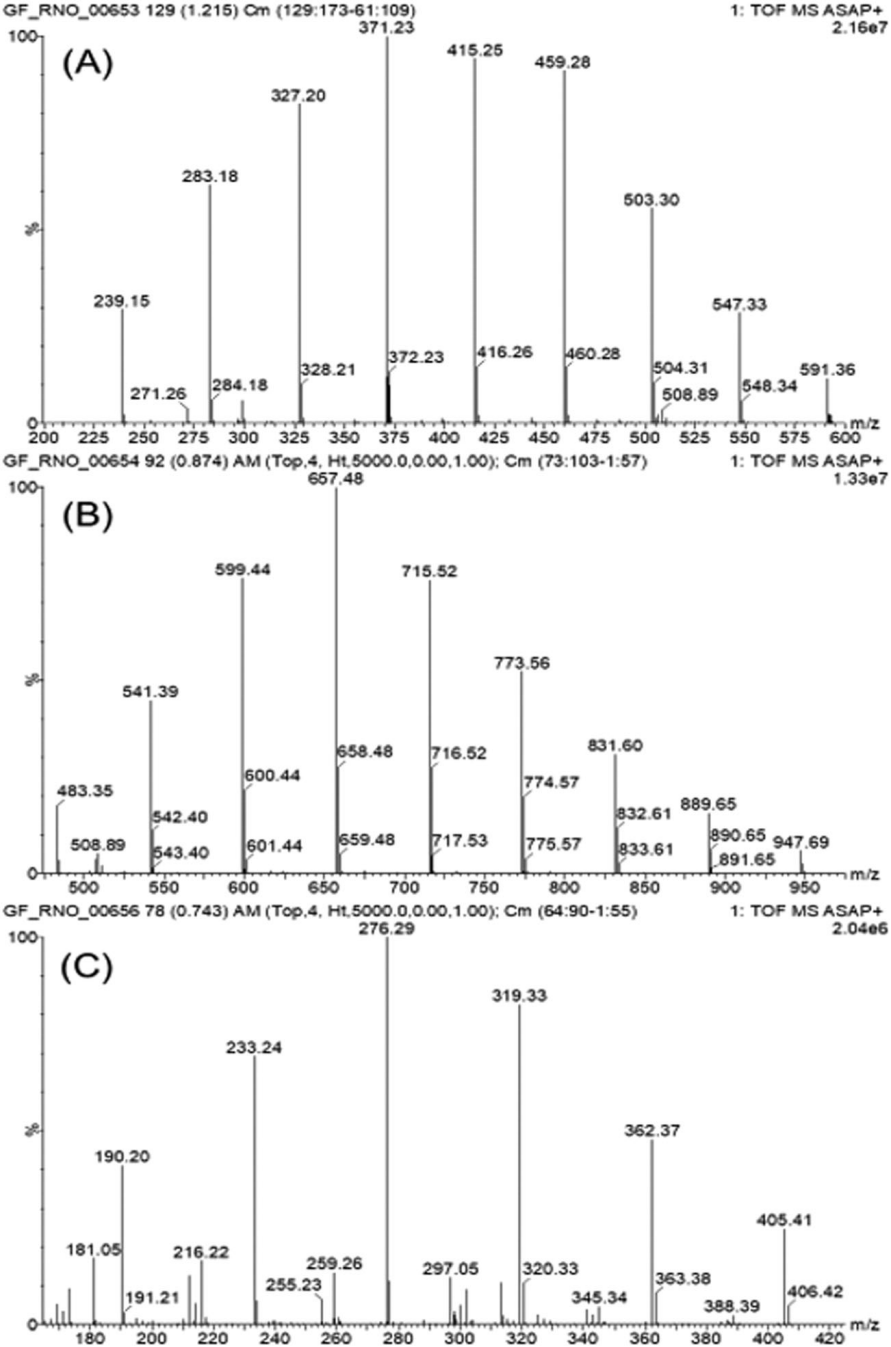
Mass spectra of low‐molecular‐mass polymers obtained using GlowFlow mass spectrometry. (A) PEG 400, *m*/*z* 239.15 to 591.36 with repeat unit *δ* 44.03. (B) PPG 725, *m*/*z* 483.35 to 947.69 with repeat unit *δ* 58.04. (C) PEI, *m*/*z* 190.20 to 405.41 with repeat unit *δ* 43.04

Three low‐average‐molecular‐mass polymers, namely PEG 400, PPG 725 and PEI, were selected for analysis using GFI mass spectrometry on the Xevo G2‐S instrument in full‐scan mode using the GlowFlow source. The samples were prepared at concentrations of 1 μg μL^−1^, and 1 μL was syringed on the end of a glass capillary before being introduced into the source using a solids analysis probe. Intense protonated ions for all three polymers were observed where the repeat units can be seen for each polymer, 44.03 for PEG 400, 58.04 for PPG 725 and 43.04 for PEI, in Figure 2. Using high‐resolution mass spectrometry, the accurate mass measurement was recorded of one of the PEG 400 ions at *m*/*z* 459.280, and a report was produced listing all the possible compositions within a certainty of 1.0 mDa (Table 1). Of the five possible compounds that were listed, the calculated *m*/*z* of 459.2805 (−0.5 mDa) was the only one that corresponded to the molecular formula of the protonated ion ([C_20_H_42_O_11_ + H]^+^) of PEG 400.

**TABLE 1:** Elemental composition report using accurate mass measurement of the PEG ion at *m*/*z* 459.2800, showing all possible options for an ion with elements C, H, N and O composition within 1.0 mDa. For each calculated *m*/*z* the corresponding uncertainty (*δ*) with respect to the measured *m*/*z*, the degree of unsaturation (DBE), the confidence of the isotope profile and empirical formula of the ion are listed

Calc. *m*/*z*	*δ* (mDa)	DBE	Confidence (%)	Formula
459.2800	0.0	17.5	1.83	C_33_H_35_N_2_
459.2805	−0.5	−0.5	66.13	C_20_H_43_O_11_
459.2805	−0.5	5.0	11.18	C_19_H_37_N_7_O_6_
459.2792	0.8	0.0	16.70	C_18_H_41_N_3_O_10_
459.2792	0.8	5.5	4.17	C_17_H_35_N_10_O_5_

### 3.2 Negative GlowFlow mass spectrometry of benzoic and cinnamic acids

Negative‐ion mass spectrometry is not as commonly used as the positive‐ion counterpart, because fewer compound classes undergo the process to form negative ions, such as deprotonation. Nonetheless, it can be more sensitive since there are fewer background ions generated. Five benzoic acids were selected for analysis: 3‐nitrobenzoic acid; 2,5‐dihydroxybenzoic acid; 4‐fluorobenzoic acid; pentafluorobenzoic acid; and pentafluorocinnamic acid. They are generally non‐polar in character with Log *P* values of between 1.6 and 2.4. The samples were prepared to a stock concentration of 1mM in methanol, and further diluted to working concentrations of between 5 and 500 pmol μL^−1^.

#### 3.2.1 Analysis of 4‐fluorobenzoic acid by loop‐injection GlowFlow

**FIGURE 3:**
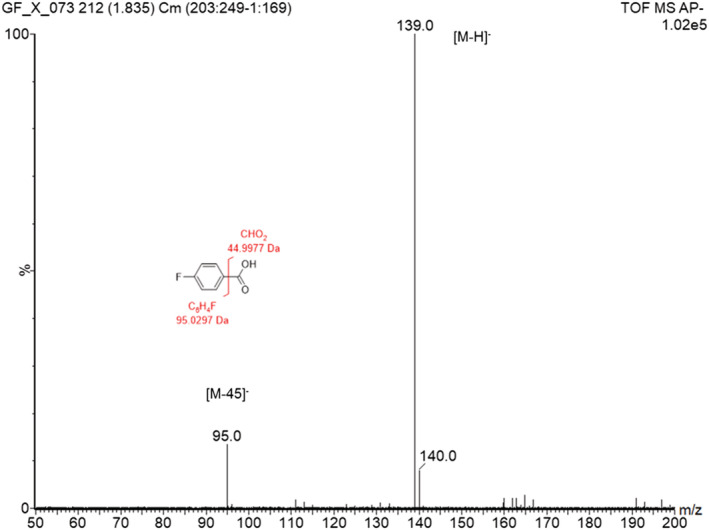
Mass spectrum of 4‐fluorobenzoic acid obtained using GlowFlow negative‐ion mass spectrometry. The base peak ion at *m*/*z* 139.0 is the deprotonated molecule [M − H]^−^. Also observed is an ion at *m*/*z* 95.0, [M − 45], possibly resulting from fragmentation occurring at the bond of the benzyl ring and CO_2_H group [Color figure can be viewed at wileyonlinelibrary.com]

The compound 4‐fluorobenzoic acid was selected and analysed at a concentration of 100 pmol μL^−1^ by loop injection using a 1 μL injection loop (14 ng total) on the Xevo G2‐S instrument in full‐scan mode. A mass spectrum is presented in Figure 3 where the base peak is the deprotonated molecule [M − H]^−^ at *m*/*z* 139.0. Also observed is an ion at *m*/*z* 95.0 at intensity 13.4% relative to the base peak, which would appear to be the result of fragmentation of the bond between the carboxyl group and the aromatic ring, resulting in the formation of a stable 4‐fluorobenzyl fragment ion.

#### 3.2.2 Separation of five benzoic acid compounds by GlowFlow LC/MS

**FIGURE 4:**
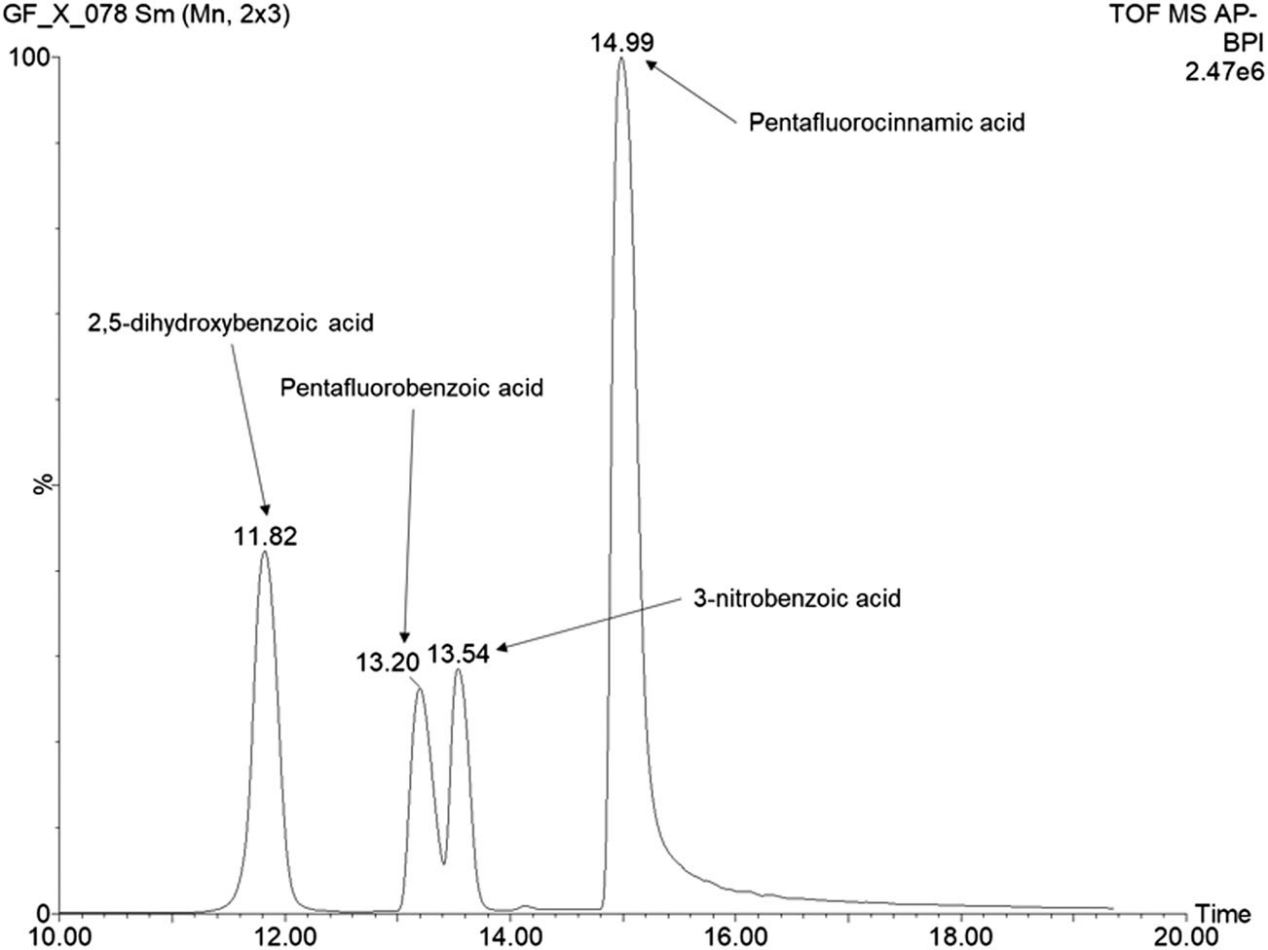
LC/MS chromatogram of five benzoic acid compounds at 100 μM (1 μL injected on column). Four of the compounds could be readily separated and identified as 2,5‐dihydroxybenzoic acid (*t*_R_ = 11*.*82 min, Log *P* = 1*.*6), pentafluorobenzoic acid (*t*_R_ = 13*.*20 min, Log *P* = 2*.*0), 3‐nitrobenzoic acid (*t*_R_ = 13*.*54 min, Log *P* = 1*.*8) and 2,3,4,5,6‐pentafluorocinnamic acid (*t*_R_ = 14*.*99 min, Log *P* = 2*.*4). The compound 4‐fluorobenzoic acid (Log *P* = 2*.*1) appeared to co‐elute at *t*_R_ = 13*.*54 min with 3‐nitrobenzoic acid and was weak in intensity (1.9% of the base peak)

To demonstrate the applicability of negative‐ion GlowFlow to LC/MS studies, the five benzoic acid compounds were separated using reverse‐phase liquid chromatography with a 1100 series HPLC coupled to a Xevo G2‐S mass spectrometer. Four of the compounds are clearly separated and identifiable in Figure 4, namely 2,5‐dihydroxybenzoic acid (*t*_R_ = 11.82 min, Log *P* = 1.6), pentafluorobenzoic acid (*t*_R_ = 13.20 min, Log *P* = 2.0), 3‐nitrobenzoic acid (*t*_R_ = 13.54 min, Log *P* = 1.8) and 2,3,4,5,6‐pentafluorocinnamic acid (*t*_R_ = 14.99 min, Log *P* = 2.4). No distinct peak can be seen for 4‐fluorobenzoic acid, and when an extracted ion chromatogram at *m*/*z* 139 is observed, it is shown to co‐elute at *t*_R_ = 13.54 min with 3‐nitrobenzoic acid with a relative intensity of only 1.9%.

The initial HPLC method provided good separation of the compounds; however, as the first compound did not elute until *t*_R_ = 11.82 min, it was not optimal in terms of maximizing efficiency. The method was therefore optimized, and the gradient conditions were adjusted as follows: 45% solvent B at 0 min, increasing linearly to 85% solvent B at 18 min, at a flow rate of 0.2 mL min^−1^.

#### 3.2.3 Analytical figures‐of‐merit for benzoic acid compounds by GlowFlow LC/MS

**FIGURE 5:**
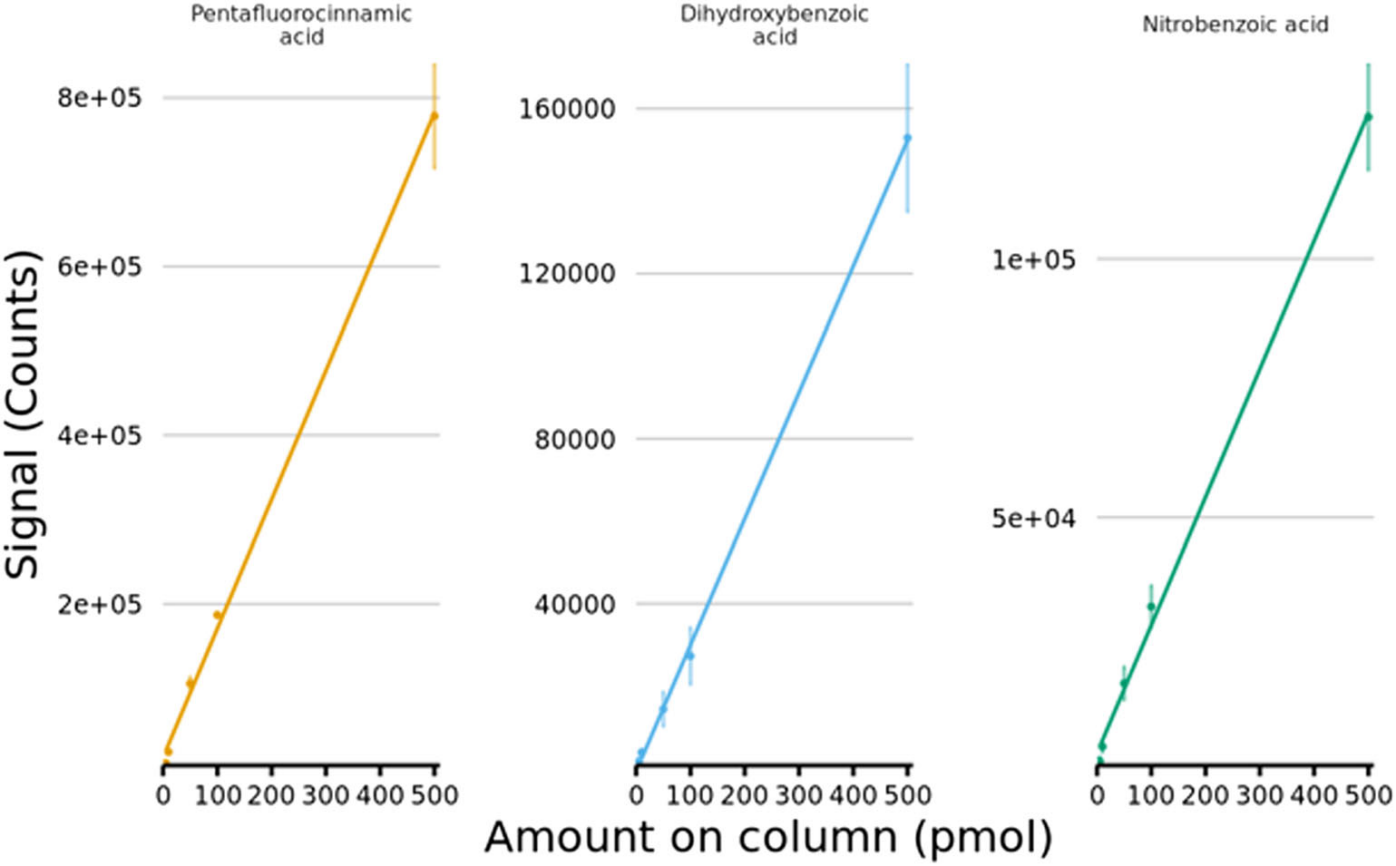
Linear regression plots of three benzoic acid compounds (*N* = 5, *n* = 3). The IDL was 436.8 fmol for pentafluorocinnamic acid (*R* ^2^ = 0.9983), 3.8 pmol for dihydroxybenzoic acid (*R* ^2^ = 0.9994) and 9.6 pmol for nitrobenzoic acid (*R* ^2^ = 0.9978) [Color figure can be viewed at wileyonlinelibrary.com]

To gain an understanding of the semi‐quantitative analytical capabilities of negative‐ion GlowFlow, an experiment using three of the benzoic acid compounds was undertaken using LC/MS. A series of five dilutions were prepared at concentrations from 5 to 500 pmol μL^−1^ (injection volume = 1 μL). The linear regression plots are presented in Figure 5. All three analytes gave a linear signal response over three orders of magnitude. The instrument detection limit (IDL)^20^ was 436.8 fmol for pentafluorocinnamic acid (*R* ^2^ = 0.9983), 3.8 pmol for dihydroxybenzoic acid (*R* ^2^ = 0.9994) and 9.6 pmol for nitrobenzoic acid (*R* ^2^ = 0.9978), which is comparable to compounds previously examined by positive‐ion GlowFlow mass spectrometry.^1^

Negative‐ion GlowFlow is capable of the analysis of compounds which typically undergo deprotonation, specifically benzoic acids. An optimized LC/MS method in negative‐ion mode has been developed to quickly analyse in less than 8 min and separate the majority of compounds (80%) in the sample. Negative‐ion GlowFlow has a limit of detection in the low picomole range for the compounds investigated with good linearity over three orders of magnitude (*R* ^2^ > 0.998).

### 3.3 Comparison of the GlowFlow source with ESI

**FIGURE 6:**
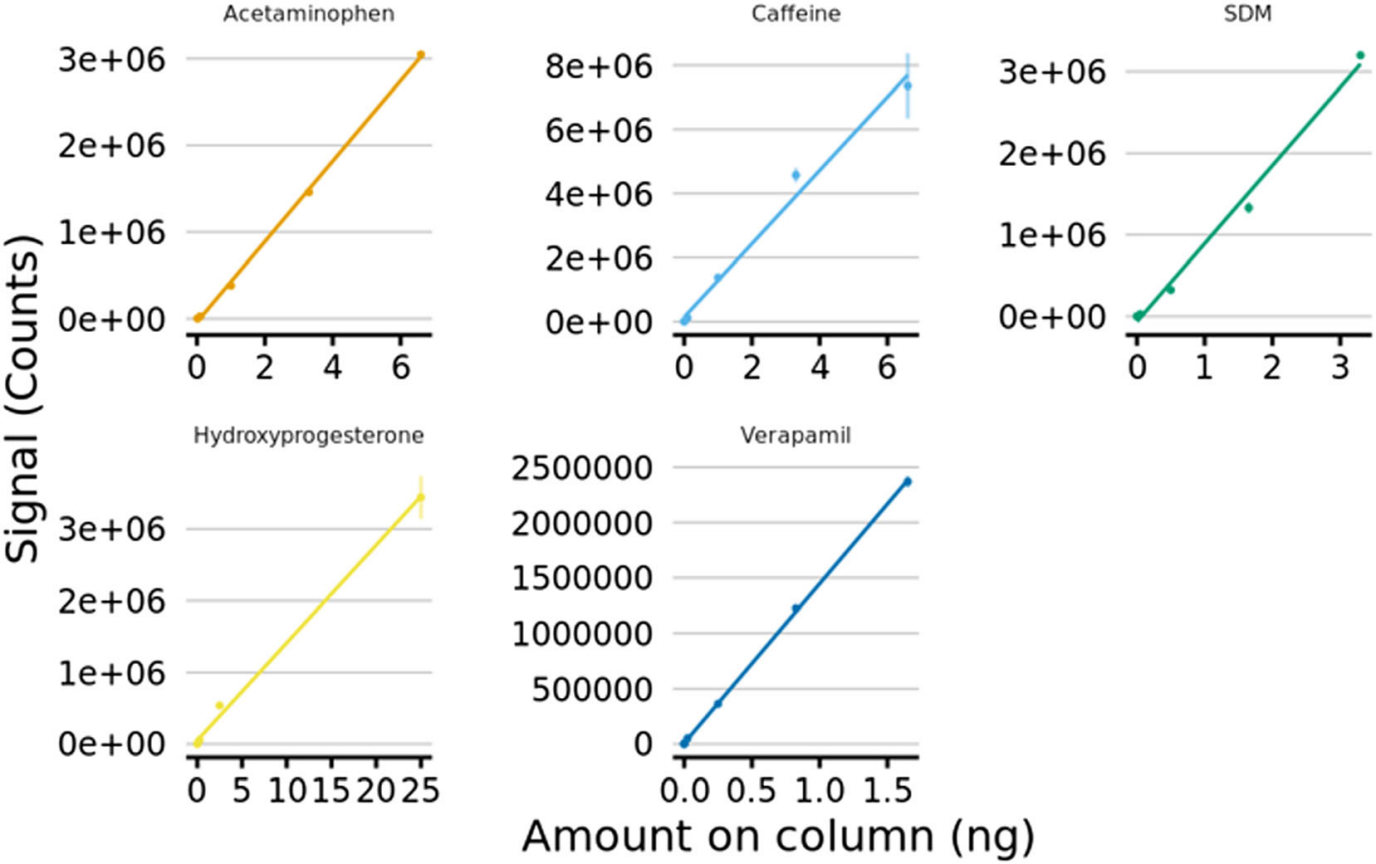
Linear regression plot of GlowFlow with a 10 μA He discharge in constant current regulation and in combination with the APCI heated nebulizer probe for sample vaporization [Color figure can be viewed at wileyonlinelibrary.com]

**FIGURE 7:**
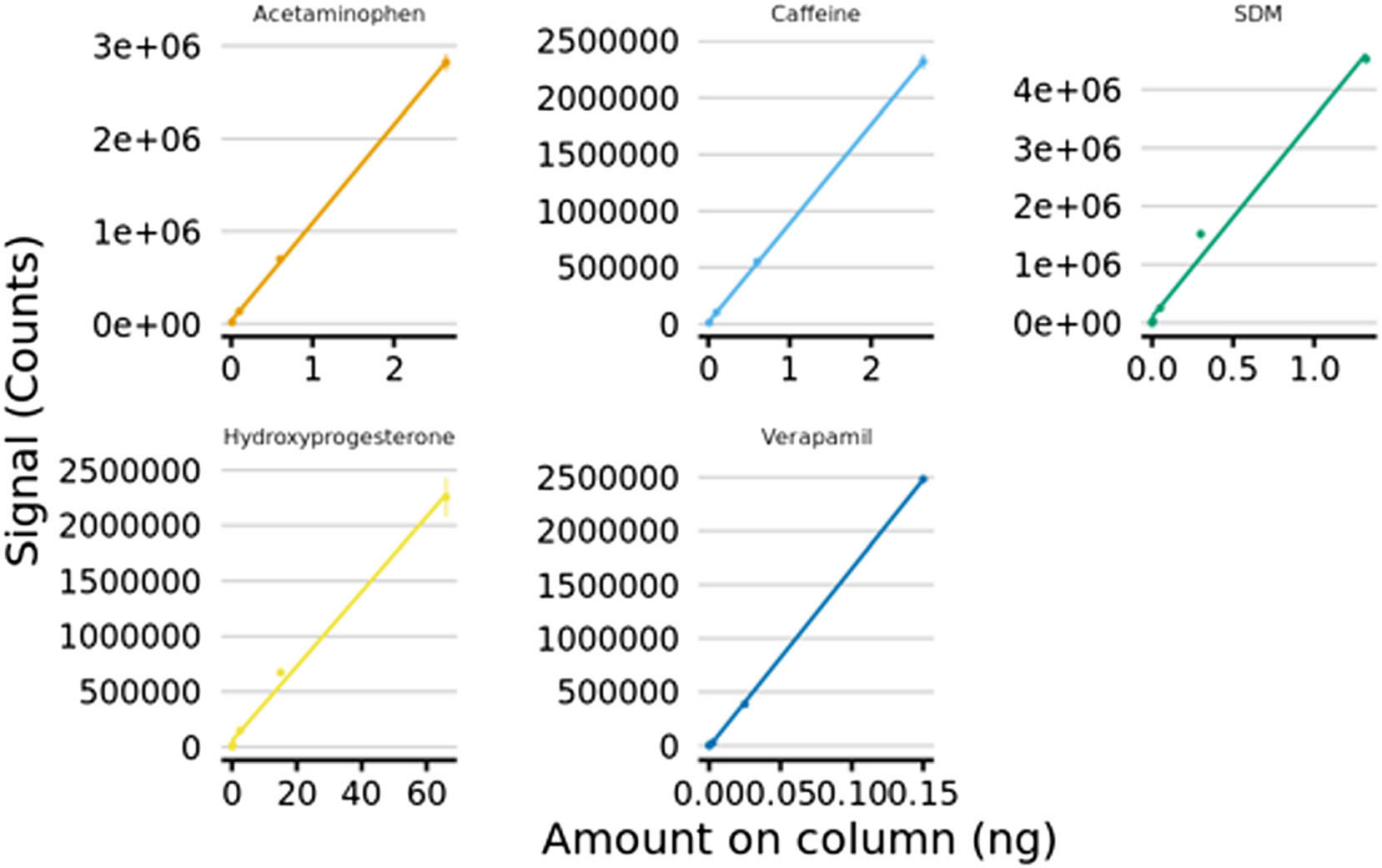
Linear regression plot of GlowFlow with a 10 μA He discharge in constant voltage regulation at 3 kV and in combination with the ESI probe (0 kV) for sample vaporization [Color figure can be viewed at wileyonlinelibrary.com]

Analysis of the ‘six‐mix solution’ containing acetaminophen, caffeine, sulfadimethoxine, hydroxyprogesterone and verapamil was undertaken using the GlowFlow ionization source on an Acquity HPLC coupled to the Xevo TQ‐S triple‐quadrupole mass spectrometer.^[22]^ The GlowFlow source was operated in two modes: firstly, with an APCI IonSABRE II (heated nebulizer) probe in combination with a 10 μA He discharge in constant current regulation; and secondly, with an ESI probe (0 kV) in combination with a 10 μA He discharge in constant voltage regulation (3 kV). The two modes introduce the samples into the source in a different manner where the APCI probe produces a heated vapour whilst the ESI probe provides a nebulized spray of liquid droplets with diameters of typically a few micrometres. The desolvation gas was set to 400 L min^−1^ and a temperature of 550°C. Data were acquired in selected reaction monitoring mode on the Xevo TQ‐S for the following transitions: acetaminophen *m*/*z* 152.0 → 109.9, caffeine 195.1 → 138.0, sulfadimethoxine 311.2 → 155.9, hydroxyprogesterone 331.2 → 109.0 and verapamil 455.3 → 165.0. Linear regression analysis for each of the five compounds was conducted in both configurations (Figures 6 and 7) and a summary of the data is presented in Tables 2 and 3 for the GlowFlow ionization source in combination with the APCI probe (*n* = 3) and ESI probe (*n* = 2), respectively.

**TABLE 2:** Summary of the results for six‐mix compounds using the GlowFlow ionization source in combination with the APCI probe. Listed are the linear dynamic range (1 d.p.), the coefficient of variability (*R* ^2^), the lowest detectable amount on column, the corresponding standard deviation (SD) at the lowest amount on column and the calculated instrument detection limit (IDL)

Compound	Linear dynamic range	*R* ^2^	Lowest amount on column (pg)	SD	IDL (fmol)
Acetaminophen	2.7	0.9986	10.00	285.3	12.44
Caffeine	3.7	0.9870	1.00	460.7	6.23
Sulfadimethoxine	3.7	0.9935	0.50	60.1	0.61
Hydroxyprogesterone	4.0	0.9969	2.50	170.5	11.35
Verapamil	4.7	0.9997	0.03	21.8	0.110.05

**TABLE 3:** Summary of the results for six‐mix compounds using the GlowFlow ionization source in combination with the ESI probe (0 kV). Listed are the linear dynamic range (1 d.p.), the coefficient of variability (*R* ^2^), the lowest detectable amount on column, the corresponding standard deviation (SD) at the lowest amount on column and the calculated instrument detection limit (IDL)

Compound	Linear dynamic range	*R* ^2^	Lowest amount on column (pg)	SD	IDL (fmol)
Acetaminophen	2.3	0.9997	10.00	4534.7	84.88
Caffeine	2.3	0.9999	10.00	192.3	3.40
Sulfadimethoxine	4.3	0.9880	0.05	27.6	0.06
Hydroxyprogesterone	4.3	0.9951	25.00	23.3	6.26
Verapamil	3.6	0.9998	0.03	221.3	0.09

The data indicate that in general, the GlowFlow ionization source is capable of routine detection of compounds at on‐column amounts in the low picograms with high linearity (*R* ^2^ > 0.98) over 2.3 and 4.7 orders of magnitude dynamic range. The results demonstrate that the performance of the ionization source is compound specific. For acetaminophen and caffeine, which are both polar (Log *P* < 1), and for non‐polar hydroxyprogesterone, there was no detectable signal below 10, 1 and 2.5 pg, respectively. Whereas for sulfadimethoxine and verapamil, the ionization source was capable of detection at on‐column amounts of 50 fg or less. The IDL for sulfadimethoxine was lower by an order of magnitude (from 612 amol to 64 amol) when using the ESI probe as the sample introductory method, and there were also marginal improvements in the IDLs for caffeine, hydroxyprogesterone and verapamil. In contrast, an improvement to the IDL was observed for acetaminophen (from 84.88 to 12.44 fmol) when using the APCI heated nebulizer probe as the sample introduction method. One reason for the GlowFlow source's poorer sensitivity to acetaminophen, with respect to the other compounds in this study, could be explained by the presence of the hydroxyl group on the phenyl ring. Previous experiments^1^, ^6^ have shown the propensity for alcohol‐ or hydroxyl‐containing compounds to lose the OH group by the well‐established mechanism of forming a stable [M − 17]^+^ ion. Analysis of the six‐mix solution by ESI was also undertaken and compared at the equivalent of 1 pg on column of sulfadimethoxine in positive‐ion mode using selected reaction monitoring mode (Figure S1). The GlowFlow ionization source is at least an order of magnitude less sensitive compared to the standard ESI method for these specific compounds.

### 3.4 Comparison of ionization sources for the detection of extractables and leachables (E&L)^21^

Analysis of 18 compounds which are commonly detected as E&L in plastics was undertaken with an Acquity HPLC coupled to the Xevo TQ‐S triple‐quadrupole mass spectrometer. Details of the compounds including structures are included in Table S3. The GlowFlow ionization source was operated with a heated nebulizer probe and a He discharge current of 10 μA in constant current regulation and the Glow Flow source was compared to ESI and APCI with a corona discharge current of 2 μA. The mass spectrometer was operated in full‐scan mode with polarity switching (*m*/*z* 50–1300 in 0.5 s, 20 ms inter‐scan delay) to gather data on positive and negative ions simultaneously. Three measurements were undertaken with volumes of 1, 5 and 10 μL injected via the HPLC system using a water (0.1% formic acid, 1mM ammonium acetate) and methanol gradient over 15 min.

#### 3.4.1 Positive‐ion mode

**FIGURE 8:**
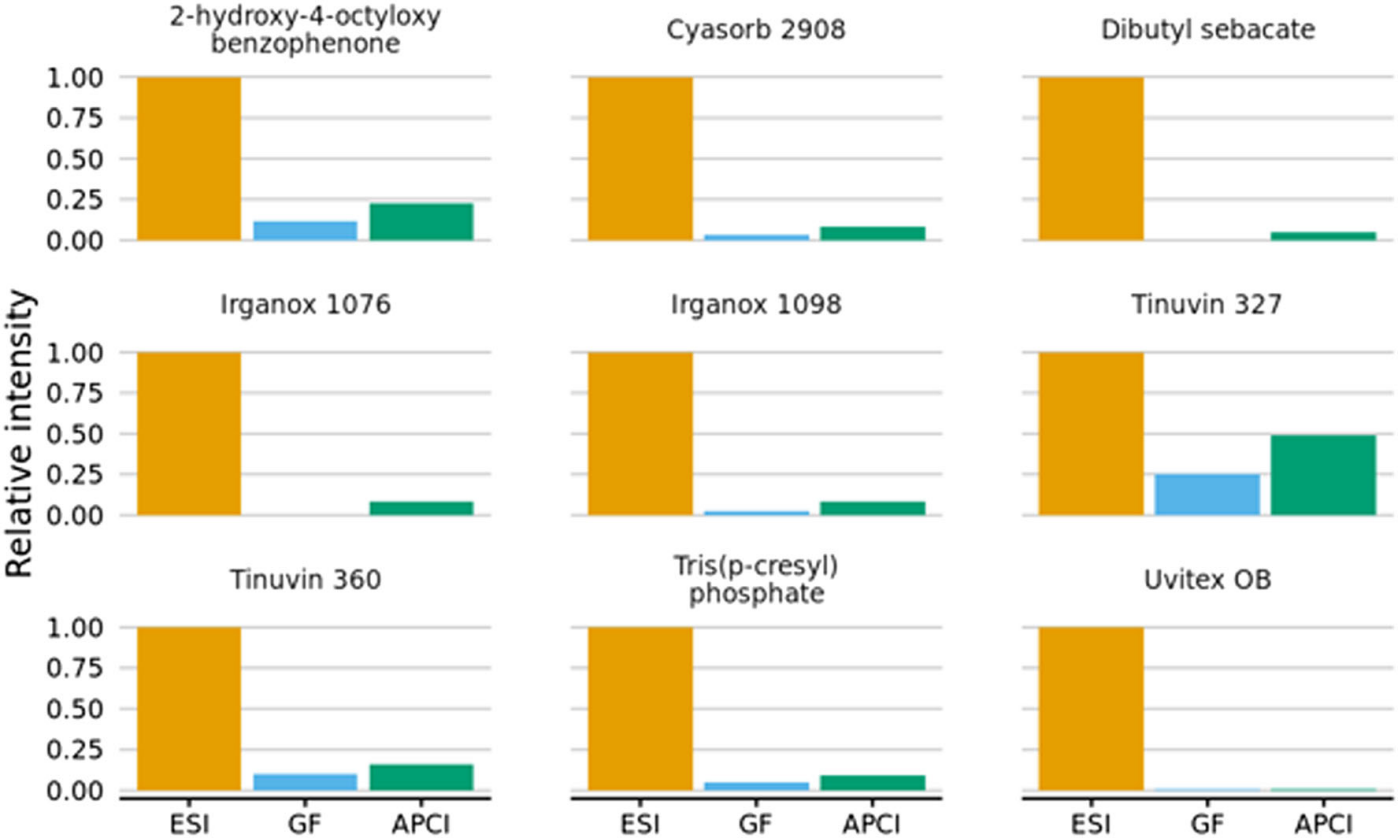
Histogram showing the relative intensities of the [M + H]^+^ ion normalized to 1.00 for ESI. The ESI source outperforms both GlowFlow and APCI in positive‐ion mode [Color figure can be viewed at wileyonlinelibrary.com]

**FIGURE 9:**
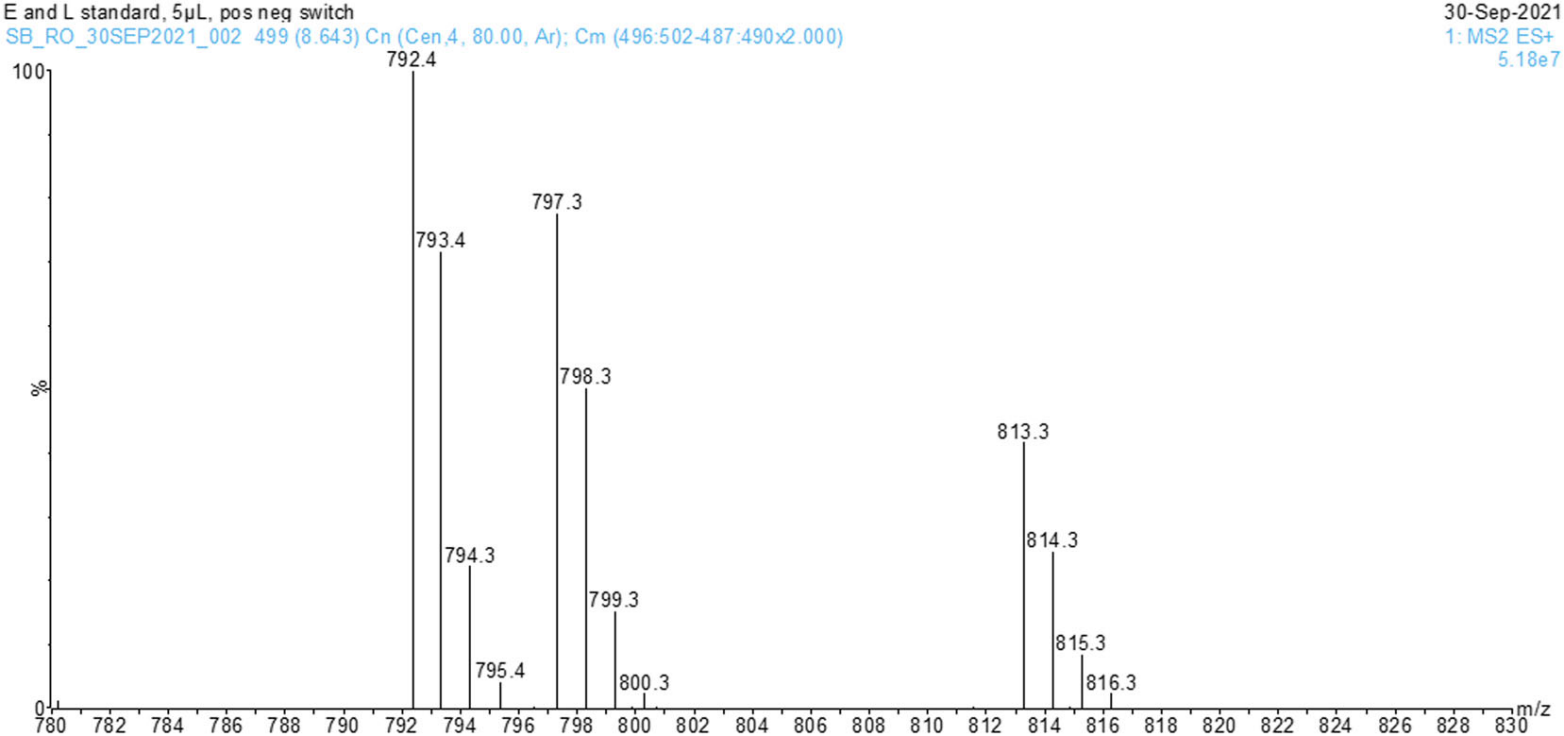
Mass spectrum of Ethanox 330 by ESI in positive‐ion mode. A series of intense ions are observed at *m*/*z* 792.4, 797.3 and 813.3 corresponding to [M + NH_4_]^+^, [M + Na]^+^ and [M + K]^+^ adducted molecular ion species. The protonated molecule is not observed [Color figure can be viewed at wileyonlinelibrary.com]

The peak area for the protonated molecule [M + H]^+^ was tabulated for the 5 μL injection and the signal intensity was normalized to the ESI data. The results for positive‐ion mode are shown in Figure 8. The GlowFlow and APCI sources both showed reduced sensitivity with respect to ESI. As is observed with the six‐mix compounds, the glow flow source is typically an order of magnitude lower in sensitivity than ESI (positive ion) under these experimental conditions. However, the data for the protonated molecules are not necessarily representative for all the compounds studied. As can be seen in Figure 9, the protonated molecule is not discernible in the mass spectrum of Ethanox 330 by ESI, while intense ions are observed for the ammonium, sodium and potassium adducted ions at *m*/*z* 792.4, 797.3 and 813.3. The number of detectable E&L compounds increased from 9 to 12 out of the 18 standards when monitoring the sodium adducts (data not shown).

Tinuvin compounds do not appear to form sodium adduct ions and there was no measurable signal. The hydroxyl group on the aromatic ring of tinuvin may be sterically hindered due to the presence of an *ortho* substituent making sodium adduction difficult. This would suggest that formation of sodium adducts may be preferred when a suitable heteroatom for bonding exists and is not sterically hindered. However, the formation of [M + Na]^+^ ions does not appear to readily occur in either GlowFlow or APCI, both of which used the APCI heated nebulizer probe to nebulize the sample. This difference is due to the different mechanism that forms the ions in the gas phase when compared to the solvated phase for ESI.^21^

#### 3.4.2 Negative‐ion mode

**FIGURE 10:**
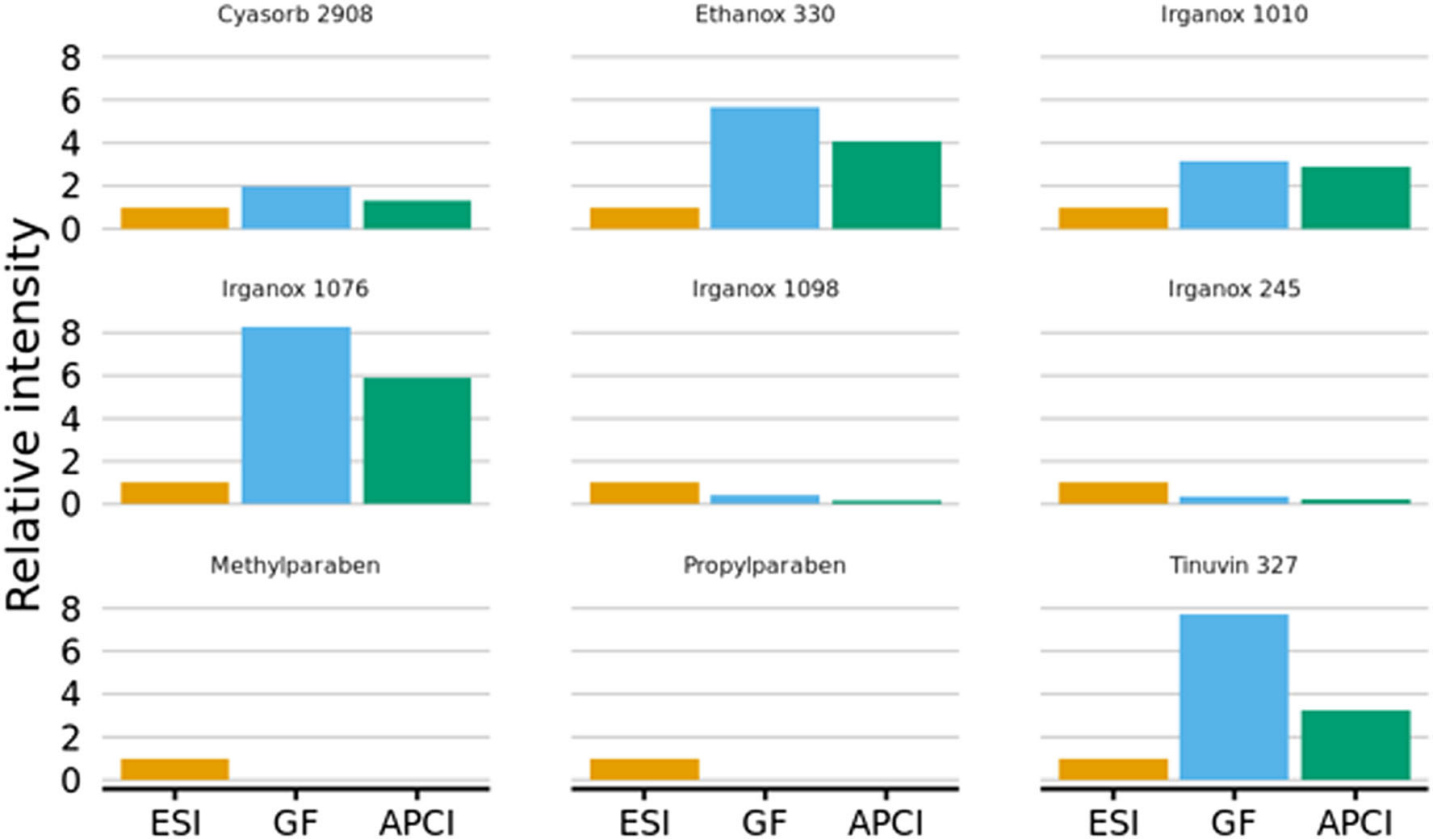
Histogram showing the relative intensities of the [M − H]^−^ ion normalized to 1.00 for ESI. For five of the nine compounds analysed, there is higher sensitivity for the GlowFlow and APCI, and higher sensitivity for four of the nine samples for ESI [Color figure can be viewed at wileyonlinelibrary.com]

In negative‐ion mode, a similar number of the compounds proved to be ionizable, with only 9 of the 18 E&L analytes giving a response under the current experimental conditions. The peak area for the deprotonated molecule [M − H]^−^ was tabulated for the 5 μL injection and the signal intensity was normalized to the ESI data. The results for negative‐ion mode are shown in Figure 10. In stark contrast to the positive‐ion data, ESI gave the highest sensitivity for only four out of the nine compounds detected, while five out of the nine compounds showed higher signal intensities with the APCI and GlowFlow when compared to ESI. The GlowFlow ionization source provided the highest relative signal response for three of the compounds, namely Cyasorb 2908, Tinuvin 327 and Irganox 1076, being 1.95, 7.74 and 8.29 times more intense than for ESI, respectively.

### 3.5 Analysis of APGC standard by direct infusion

The APGC standard containing eight compounds (see Table S2) was analysed using the direct infusion method at a flow rate of 20 μL min^−1^ and the compounds were initially detected in full‐scan mode with the Xevo TQ‐S triple‐quadrupole mass spectrometer. The ions observed are recorded in Table 4. Five of the potential compounds were identified by their *m*/*z* values using the GlowFlow source; however the ion at *m*/*z* 179.1 could represent either phenanthrene or anthracene or more likely both. ESI performed poorly with this set of compounds where only a weak ion at *m*/*z* 179 was observed; the ion of benzo[*ghi*]perylene may also have tentatively been observed.

**TABLE 4:** List of compounds from the APGC standard detected by GlowFlow and ESI. The *m*/*z* is recorded along with the corresponding ion species

Compound	GlowFlow *m*/*z*	ESI *m*/*z*
1,2‐Dichlorobenzene	—	—
Phenanthrene	179.1 [M + H]^+^	179.1 [M + H]^+^
Anthracene	179.1 [M + H]^+^	179.1 [M + H]^+^
Octafluoronaphthalene	—	—
Benzo[*ghi*]perylene	277.1 [M + H]^+^	—
Hexachlorobenzene	—	—
2,3,7,8‐Tetrachlorodibenzo‐*p*‐dioxin	319.7 [M]^+^	—
Endosulfan	404.7 [M + H]^+^	—

**FIGURE 11:**
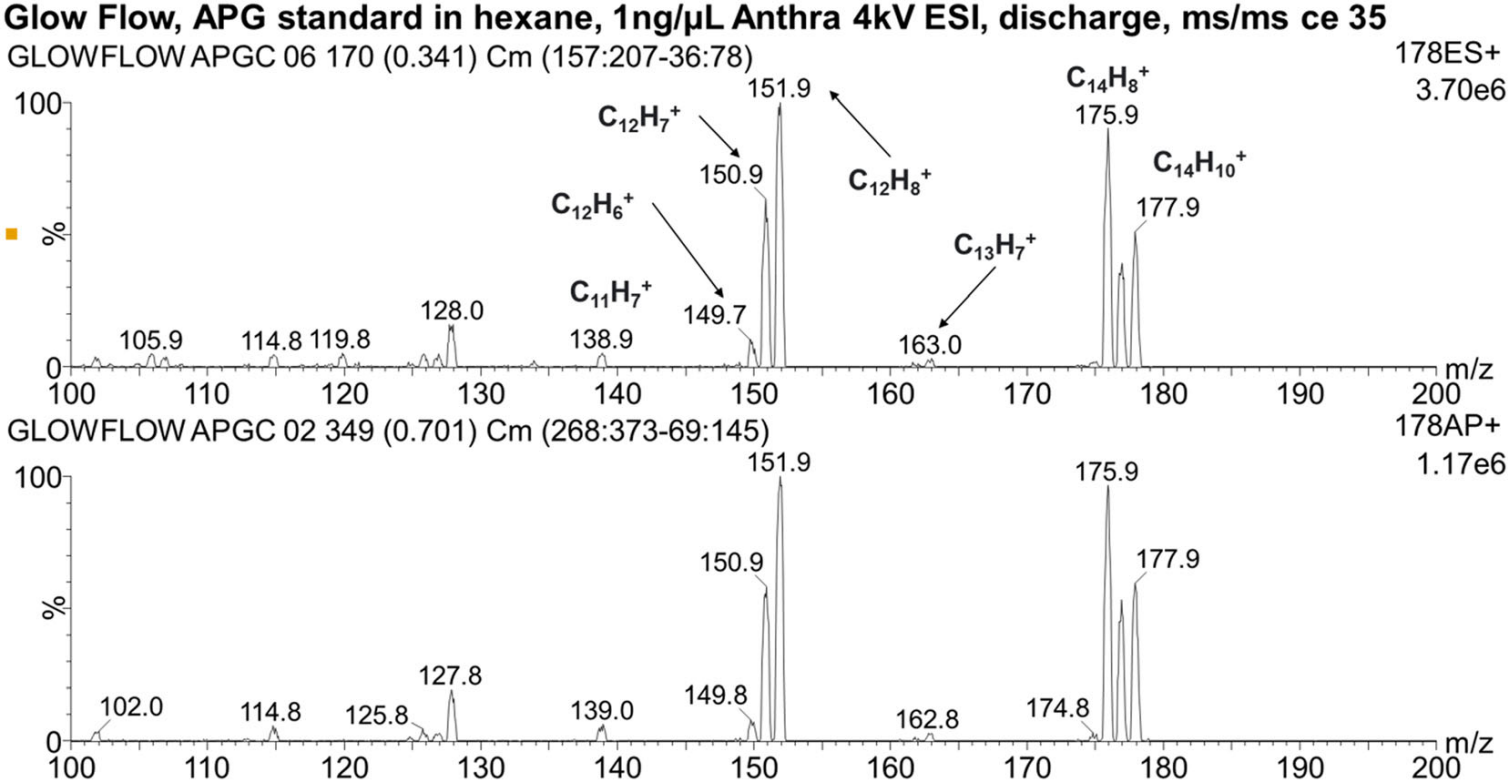
MS/MS mass spectrum of the fragment ions for the precursor ion at *m*/*z* 178. Top panel: ESI; bottom panel: GlowFlow. The collision energy was set to 35 eV [Color figure can be viewed at wileyonlinelibrary.com]

**FIGURE 12:**
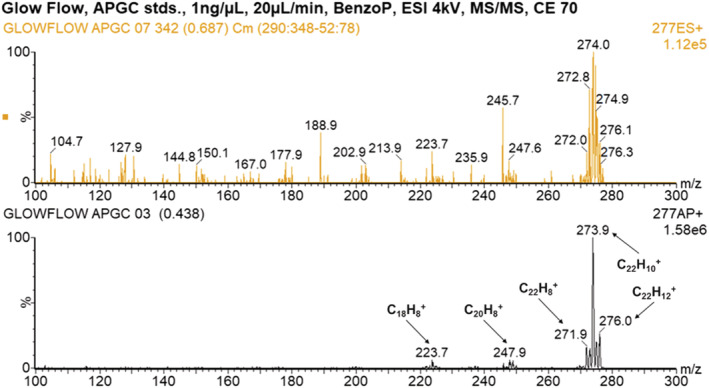
MS/MS mass spectrum of the fragment ions for the precursor ion at *m*/*z* 277 of benzo[*ghi*]perylene. Top panel: ESI; bottom panel: GlowFlow. The collision energy was set to 70 eV [Color figure can be viewed at wileyonlinelibrary.com]

**SCHEME 1:**
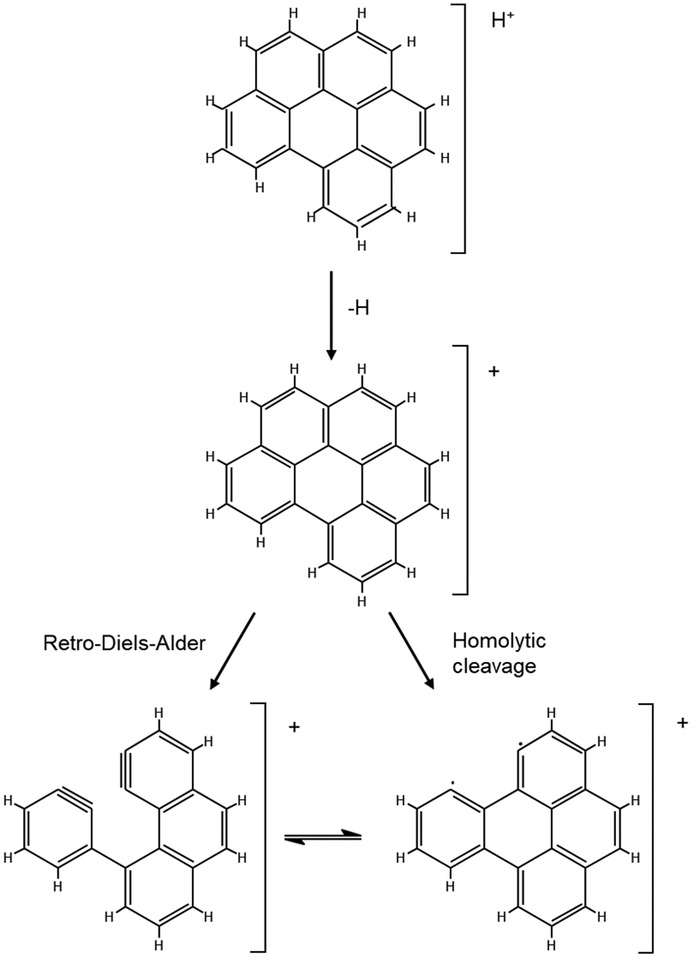
Proposed cleavage diagram of the protonated molecule of benzo[*ghi*]perylene. Two possible mechanisms for fragmentation of polyaromatic ring structure were proposed by Wang et al^22^

Further analysis was undertaken using MS/MS. A collision energy of 35 eV was used to fragment ions of the precursor ion at *m*/*z* 178 for the phenanthrene/anthracene compound. Observed are fragments at *m*/*z* 177, 176 and 175, corresponding to the sequential loss of hydrogen from the precursor ion. Also observed are the fragments 175, 162, 149, a transition of *m*/*z* = 13 equivalent to the loss of CH, and fragments 177, 151, 138, a transition of *m*/*z* = 26 equivalent to the loss of C_2_H_2_. The fragmentation patterns for both GlowFlow and ESI are similar, and no noticeable differences are observed (Figure 11). A MS/MS study was also conducted for the benzo[*ghi*]perylene compound (collision energy of 70 eV) and the fragments of the precursor ion at *m*/*z* 277 were recorded in Figure 12. The signal for GlowFlow is an order of magnitude greater than that for ESI for the fragment ions. A series of fragment ions 276, 275, 274, 273 and 272, equivalent to the loss of hydrogen atoms from the precursor ion, are observed. Also observed are fragment ions 274, 248 and 222, a transition of *m*/*z* = 26, equivalent to the loss of C_2_H_2_. For both compounds examined, this would appear to be the result of fragmentation of the ring structure (Scheme 1).

## 4 CONCLUSIONS

GFI appears to be a versatile ionization technique, with potentially high sensitivity in some applications. The technique is currently not widely available and its potential sensitivity and range of application are generally unknown in the field of organic mass spectroscopy. It is generally an easy technique to fit to API systems and the GlowFlow design is particularly simple to retrofit due to its small size. GFI's sensitivity is in the picomole to femtomole range; however, we intend to further research the GlowFlow design with a dopant bleed and hope to approach the sensitivities exhibited by an ESI source. The GlowFlow design exhibits a fast response making it compatible with ultrahigh‐performance liquid chromatography. GFI in broad terms has considerable potential for development with small‐molecule work (under around 800 Da). Due to its small size, it can be mounted and used alongside other API sources and thus be readily switched in and out of operation without source removal and provide added value to an analyst's tools. There have been only limited studies with higher mass compounds since it is currently insensitive at higher mass.

These data have shown GlowFlow ionization to be an order of magnitude less sensitive than ESI in positive‐ion mode, but nevertheless, it does exhibit a limit of detection in the low nanogram to femtogram range, with values of *R* ^2^ > 0.98 over two to four orders of magnitude. The GlowFlow ionization source provided signal intensity of an order of magnitude greater than that of ESI for the APGC standard mix and ionized several compounds the ESI could not. The high and positive Log *P* values for the APGC compounds (Table S2) suggest that GlowFlow is particularly suited for the analysis of more non‐polar compounds, which is consistent with historical observations of ionization sources that are based on discharges.^6^ A particular strength of GlowFlow was demonstrated in negative‐ion mode in the analysis of E&L compounds commonly used in industry as polymer additives and preservatives, offering between 1.95 and 8.29 times enhancement of the signal when compared to ESI. Though GlowFlow will not displace existing ionization sources, it may prove to be an important addition as part of a multimodal ionization source, giving complementary data on compounds less amenable to ESI, in a step towards the development of a universal ionization source.

### PEER REVIEW

The peer review history for this article is available at https://publons.com/publon/10.1002/rcm.9327.

## Supporting information


**Table S1.** List of the five compounds in the “Six‐mix” standard. The monoisotopic mass (4 d.p.), the concentration of the compounds in the stock solution and the Log P values are listed for reference.
**Table S2.** List of the eight compounds in the APGC standard. The monoisotopic mass (4 d.p.), the concentration of the compounds in the stock solution and the Log P values are listed for reference.
**Table S3.** List of the 18 compounds in the extractables & leachables screening standard. The monoisotopic mass (4 d.p.), the concentration of the compounds in the stock solution and the Log P values are listed for reference.
**Figure S1.** Histogram of ESI and GlowFlow signal intensities. Relative intensity of GlowFlow in comparison to the normalised ESI signal intensity for the 6‐mix sample in positive ion mode.

## Data Availability

The data that support the findings of this study are available from the corresponding author upon reasonable request.
