# Supplementary material for: Glow flow ionization mass spectrometry of small molecules. A comparison of a glow flow ionization source (‘GlowFlow’) with electrospray ionization and atmospheric pressure chemical ionization
Source: Rapid Commun Mass Spectrom. 2022 Jun 15;36(15):e9327. doi: 10.1002/rcm.9327 (PMC9285393; doi:10.1002/rcm.9327)
Supplement: Supplementary file 1 — Table S1. List of the five compounds in the “Six‐mix” standard. The monoisotopic mass (4 d.p.), the concentration of the compounds in the stock solution and the Log P values are listed for reference. Table S2. List of the eight compounds in the APGC standard. The monoisotopic mass (4 d.p.), the concentration of the compounds in the stock solution and the Log P values are listed for reference. Table S3. List of the 18 compounds in the extractables & leachables screening standard. The monoisotopic mass (4 d.p.), the concentration of the compounds in the stock solution and the Log P values are listed for reference. Figure S1. Histogram of ESI and GlowFlow signal intensities. Relative intensity of GlowFlow in comparison to the normalised ESI signal intensity for the 6‐mix sample in positive ion mode. [file RCM-36-0-s001.docx]

**SUPPLEMENTARY INFORMATION**

**Glow flow ionization (GFI) mass spectrometry of small molecules. A comparison of a glow flow ionization source “GlowFlow” to electrospray ionization (ESI) and atmospheric pressure chemical ionization (APCI).**

Rhodri N. Owen^1^, Stevan Bajic^2^, Steven L. Kelly^1^, Michael R. Morris^2^ and A. Gareth Brenton^1^

^1^Institute of Life Science, Faculty of Medicine, Health and Life Science, Swansea University, Singleton Park, Swansea, UK.

^2^Waters Corp., Wilmslow, UK.

| Compound | Formula | Monoisotopic  Mass (Da) | Stock Conc | Log P |
| --- | --- | --- | --- | --- |
| Acetaminophen | C_8_H_9_NO_2_ | 151.0633 | 2 ng/µL | 0.5 |
| Caffeine | C_8_H_10_N_4_O_2_ | 194.0804 | 2 ng/µL | -0.1 |
| Sulfadimethoxine | C_12_H_14_N_4_O_4_S | 310.0736 | 1 ng/µL | 1.6 |
| Verapamil | C_27_H_38_N_2_O_4_ | 454.2832 | 0.5 ng/µL | 3.8 |
| Hydroxyprogesterone | C_21_H_30_O_3_ | 330.2195 | 50 ng/µL | 3.2 |

**Table S1.** List of the five compounds in the “Six-mix” standard. The monoisotopic mass (4 d.p.), the concentration of the compounds in the stock solution and the Log P values are listed for reference.

| Compound | Formula | Monoisotopic  Mass (Da) | Stock Conc | Log P |
| --- | --- | --- | --- | --- |
| 2,3,7,8 Tetrachloro-dibenzo-p-dioxin | C_12_H_4_Cl_4_O_2_ | 319.8965 | 1ng/µL | 6.4 |
| Phenanthrene | C_14_H_10_ | 178.0783 | 1ng/µL | 4.5 |
| Hexachlorobenzene | C_6_Cl_6_ | 281.8131 | 1ng/µL | 5.7 |
| Octafluoronaphthalene | C_10_F_8_ | 271.9872 | 1ng/µL | 4.0 |
| 1,2-Dichlorobenzene | C_6_H_4_Cl_2_ | 145.9690 | 1ng/µL | 3.4 |
| Anthracene | C_14_H_10_ | 178.0783 | 1ng/µL | 4.4 |
| Endosulfan | C_9_H_6_Cl_6_O_3_S | 403.8169 | 1ng/µL | 3.8 |
| Benzo[*ghi*]perylene | C_22_H_12_ | 276.0939 | 1ng/µL | 6.6 |

**Table S2.** List of the eight compounds in the APGC standard. The monoisotopic mass (4 d.p.), the concentration of the compounds in the stock solution and the Log P values are listed for reference.

| Compound | Formula | Monoisotopic  Mass (Da) | Stock Conc | Log P |
| --- | --- | --- | --- | --- |
| Methylparaben  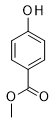 | C_8_H_8_O_3_ | 152.0473 | 100 ppb | 1.88 |
| Propylparaben  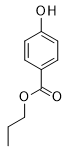 | C_10_H_12_O_3_ | 180.0786 | 100 ppb | 2.90 |
| Diethyl phthalate  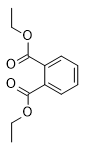 | C_12_H_14_O_4_ | 222.0892 | 100 ppb | 2.71 |
| Tinuvin P  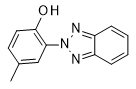 | C_13_H_11_N_3_O | 225.0902 | 1 ppm | 4.31 |
| Dibutyl sebacate  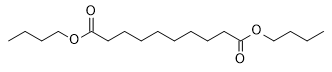 | C_18_H_34_O_4_ | 314.2457 | 100 ppb | 5.96 |
| Diphenyl phthalate  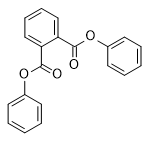 | C_20_H_14_O_4_ | 318.0892 | 100 ppb | 3.57 |
| 2-hydroxy-4-octyloxy benzophenone  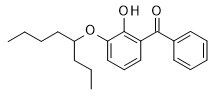 | C_21_H_26_O_3_ | 326.1882 | 100 ppb | 7.56 |
| Tinuvin 327  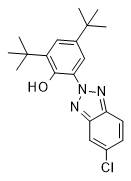 | C_20_H_24_ClN_3_O | 357.1608 | 1 ppm | 7.54 |
| Tris(p-cresyl) phosphate  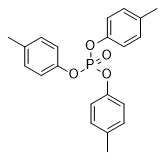 | C_21_H_21_O_4_P | 368.1177 | 100 ppb | 5.11 |
| Uvitex OB  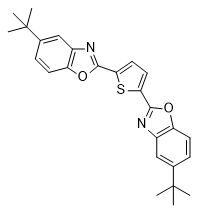 | C_26_H_26_N_2_O_2_S | 430.1715 | 100 ppb | 7.22 |
| Cyasorb 2908  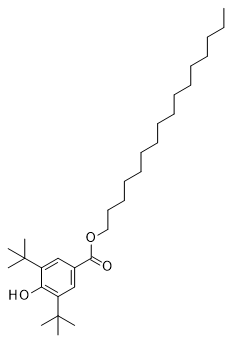 | C_31_H_54_O_3_ | 474.4073 | 100 ppb | 12.48 |
| Irganox 1076  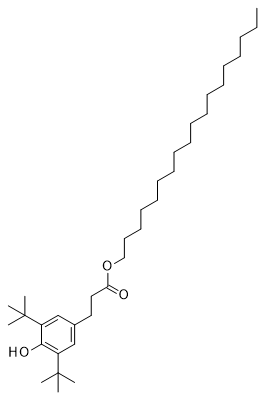 | C_35_H_62_O_3_ | 530.4699 | 1 ppm | 13.53 |
| Irganox 245  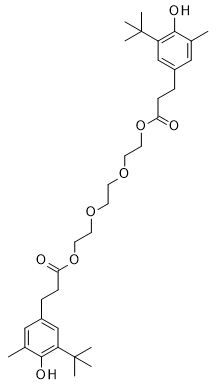 | C_34_H_50_O_8_ | 586.3506 | 100 ppb | 7.61 |
| Irganox 1098  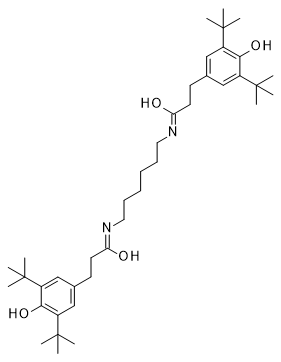 | C_40_H_64_N_2_O_4_ | 636.4866 | 100 ppb | 9.82 |
| Tinuvin 360  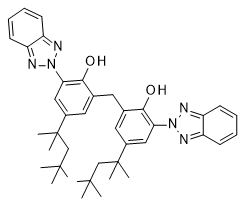 | C_41_H_50_N_6_O_2_ | 658.3995 | 1 ppm | 14.48 |
| Ethanox 330  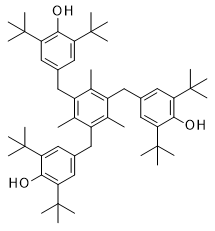 | C_54_H_78_O_3_ | 774.5951 | 1 ppm | 16.33 |
| Uvinul 3030  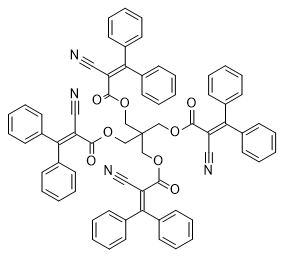 | C_69_H_48_N_4_O_8_ | 1060.3472 | 100 ppb | 12.97 |
| Irganox 1010  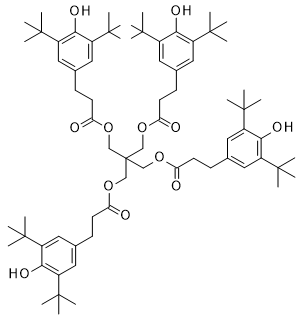 | C_73_H_108_O_12_ | 1176.7841 | 1 ppm | 18.83 |

**Table S3.** List of the 18 compounds in the extractables & leachables screening standard. The monoisotopic mass (4 d.p.), the concentration of the compounds in the stock solution and the Log P values are listed for reference.


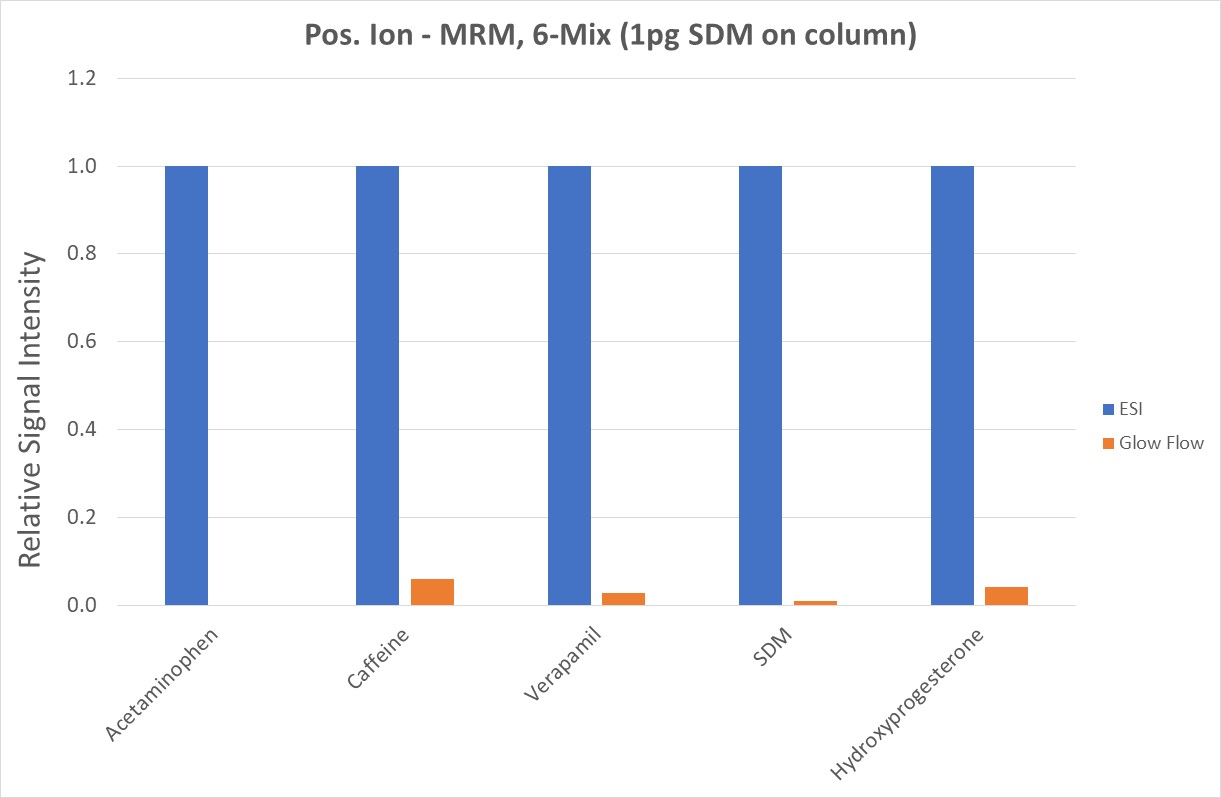


**Figure S1.** Histogram of ESI and GlowFlow signal intensities. Relative intensity of GlowFlow in comparison to the normalised ESI signal intensity for the 6-mix sample in positive ion mode.
